# Relationships between the knee injury and osteoarthritis outcome score (KOOS) and smartphone ecological momentary assessment outcomes in people with knee osteoarthritis

**DOI:** 10.1016/j.ocarto.2026.100809

**Published:** 2026-05-01

**Authors:** Mark Overton, Jennifer Dunn, Deborah Snell, Ramakrishnan Mani

**Affiliations:** a Graduate School of Health, University of Technology Sydney, Sydney, Australia; b Department of Orthopaedic Surgery and Musculoskeletal Medicine, University of Otago, Christchurch, New Zealand; c School of Physiotherapy, University of Otago, Dunedin, New Zealand

**Keywords:** Knee osteoarthritis, pain, Ecological momentary assessment, Measurement properties, Knee injury and osteoarthritis outcome score, Outcome measure

## Abstract

**Introduction:**

The Knee Injury and Osteoarthritis Outcome Score (KOOS) is a recommended recall-based self-report questionnaire assessing pain and function in people with knee osteoarthritis (OA). Smartphone Ecological Momentary Assessment (EMA) could be a more accurate method to assess current symptoms during everyday life. Whether outcomes collected via smartphone EMA align with recall-based patient-reported outcome measures (PROMs), such as the KOOS, is yet to be explored. Therefore, this study aims to explore relationships between outcomes collected via smartphone EMA and the KOOS in people with knee OA. Secondary aims include exploring whether relationships are maintained after adjusting for demographic and clinical factors influencing knee OA outcomes.

**Methods:**

A smartphone EMA survey was developed, piloted and then administered to people with knee OA. Participants were asked to rate current symptoms (i.e. pain intensity, pain interference, pain bothersomeness and stiffness) three times daily at random times during the morning, afternoon and evening for two weeks. Following this, participants completed a KOOS to collect pain, symptoms, function (daily living, sport and recreation) and quality of life outcomes. Bivariate correlations were performed to explore relationships between outcomes collected via smartphone EMA and the KOOS. Partial correlations were then performed to control for age, sex, body mass index (BMI) and baseline pain intensity.

**Results:**

Eighty-six people with knee OA completed both smartphone EMA and the KOOS. There was no loss to follow-up. Statistically significant inverse relationships (ranging in strength from weak to strong) were demonstrated between pain intensity (r = −0.69, p = <0.01), interference (r = −0.62, p = <0.01), bothersomeness (r = 0.78, p = <0.01) and knee stiffness (r = 0.61, p = <0.01) measured via the two methods. After controlling for established covariates, statistically significant inverse relationships were maintained although the strength of these relationships reduced overall, ranging from weak to moderate.

**Discussion and conclusion:**

Smartphone EMA appears to be valid when compared with recommended recall-based PROMs assessing pain and function in people with knee OA, even after controlling for knee OA prognostic factors. Collecting pain and function outcomes in a momentary manner could offer greater ecological validity to living with knee OA. Additionally, EMA could help to improve our understanding of concurrent psychosocial and lifestyle factors in shaping pain and functional outcomes in people with knee OA.

## 1 Introduction

Collecting patient outcomes is an important part of the clinical assessment. The Osteoarthritis Research Society International and Outcome Measures in Rheumatology (OMERACT) groups endorse the use of patient-reported outcome measures (PROMs) in people with knee OA [[1], [2], [3], [4]]. These include outcome measures that assess pain, self-reported physical functioning and health-related quality of life [4]. The Knee Injury and Osteoarthritis Outcome Score (KOOS) is a key OARSI-recommended self-report questionnaire, which is used to assess pain, symptoms, daily living function, sport and recreation function, as well as knee-related quality of life. This measure asks people to recall and rate their symptoms and functional limitations experienced over the previous week [5,6]. However, potential limitations with PROMs, including the KOOS, are their self-reported, subjective nature and susceptibility for recall biases with patients recollecting symptoms/behaviours over time, which could potentially influence responses [7,8].

Ecological Momentary Assessment (EMA) is a self-reported assessment method that may better capture the range of symptoms and limitations involved in the knee OA pain experience [[9], [10], [11]]. A defining characteristic of EMA is that measures are repeated multiple times daily to reveal patterns of transient, fluctuating symptoms such as pain in real-time, real-life contexts [[9], [10], [11], [12], [13]]. EMA methods continue to evolve, relying less on paper diaries and more on technology, including smartphones and wearable devices. These technological advancements have further assisted with the robustness of the data collected [12,14]. Strengths of EMA include its ability to reduce recall bias and measurement errors while also offering greater ecological validity with measurement occurring in the environment and context of a person's life [9,10,13,15]. Furthermore, repeated measurements collected during EMA allow for within-person relationships to be explored in ‘real-life’ contexts [12,13].

A recent systematic review and meta-analysis, including 14 studies, explored the relationship between recalled versus momentary assessments of pain intensity and interference collected on numeric rating scales [11]. This revealed that outcomes collected via these different methods were well correlated. Therefore, support was demonstrated for the psychometric properties of EMA in the assessment of pain-related outcomes [11]. Pain conditions included in this review were diverse and included fibromyalgia, chronic back pain, irritable bowel syndrome and spinal cord injury. Some participants with OA were included; however, within mixed samples. Additionally, the methods of momentary data collection were variable, with ‘pen and paper’ methods, short message service, as well as electronic diaries being used [11].

A recent pilot study in people with knee OA found differences in pain and functional outcomes collected via EMA versus recall-based, retrospectively collected pain outcomes [8]. Specifically, higher levels of pain intensity were reported on the retrospective assessment when compared to their EMA, particularly in those who experience more pain peaks/flare-ups. Authors therefore concluded that retrospective pain outcomes are affected by recall bias [8]. However, these observations are not without limitations. This study involved an extended period of recall (more than one month), and only a simple numeric rating scale was used to collect outcomes, rather than a recommended PROM for assessing pain and function in people with knee OA. Additionally, the influence of demographic and clinical factors on knee OA outcomes, such as age, sex, and BMI, was not considered [16].

To the best of our knowledge, no study has examined whether pain and functional outcomes collected via smartphone EMA and OARSI-recommended PROMs are comparable in people with knee OA.

Therefore, this study aims to explore relationships between pain severity (pain intensity) and stiffness outcomes collected via smartphone EMA, and outcomes from equivalent KOOS subscales in people with knee OA. The secondary aims of this study are to explore relationships between pain interference and bothersomeness outcomes collected via EMA and subscales of the KOOS, as well as whether relationships are influenced by demographic, anthropometric, and clinical factors associated with knee OA outcomes.

## 2 Methods

This is a novel secondary analysis of Understanding Knee Osteoarthritis Pain Experiences (U-KOPE) data which was a prospective longitudinal study completed in 2022 in Dunedin, New Zealand [17,18]. This study was developed in consultation with the Strengthening the Reporting of Observational Studies in Epidemiology statement, the Checklist for Reporting Ecological Momentary Assessment Studies and EMA literature [[19], [20], [21], [22], [23], [24]]. Ethical approval was obtained from the Central Health and Disability Ethics Committee of New Zealand (21/CEN/89). The Consensus-based Standards for the selection of health Measurement Instruments (COSMIN) study design checklist was used when reporting the current study to ensure inclusion of recommended components for studies assessing criterion validity [25].

### 2.1 Inclusion and exclusion criteria

Participants were eligible for inclusion if they fulfilled a clinical diagnosis of knee OA based on the National Institute for Health and Care Excellence guidelines (≥45 years of age, activity-related joint pain, morning stiffness (if experienced) lasting ≤30 min) and/or reported having a diagnosis of knee OA provided by a health professional [26]. Additionally, to be eligible, participants had to report experiencing knee pain on most days for at least three months.

Participants were excluded based on self-reports of being a non-English speaker, >85 years of age, unable to use a smartphone, had other rheumatological and autoimmune conditions, had uncontrolled hypertension, skin conditions, lower limb sensory loss, had undergone or were scheduled for knee arthroplasty, were recovering from a separate leg injury, were pregnant or within six months postpartum, had a neurological condition, impaired cognition or psychiatric illness (excluding stress and mild to moderate anxiety or depression). Participants were recruited from hospital outpatient settings and the community.

### 2.2 Outcome measures

A smartphone EMA survey made up of single-item questions for constructs of interest was developed using the m-Path application before being piloted on individuals with knee OA [18,27]. Further details on the development and piloting process can be found by accessing our previously published study [18]. Primary variables of interest for this study included pain intensity, pain bothersomeness, pain interference and knee stiffness, with higher provided scores representing worse outcomes.

The KOOS was collected to assess knee symptoms and their impact on daily life [6]. This measure consists of five subscales assessing pain, symptoms, daily living function, sport and recreation function and quality of life [6]. Participants are asked to rate their experience during the last week on a 5-point Likert scale (Never, Rarely, Sometimes, Often, Always) for each item. Each subscale is then scored separately with scores ranging between 0 (extreme problems) and 100 (no problems) [6]. The KOOS has been shown to demonstrate adequate content validity, internal consistency, test-retest reliability, construct validity and responsiveness in people with knee OA [1].

### 2.3 Study procedures

Eligible participants attended a baseline assessment, then completed a two-week smartphone EMA. Following this they attended two further assessments: two weeks (immediately following EMA) and nine weeks following baseline assessment. The KOOS was collected at all timepoints.

Participant characteristics, including demographic and clinical information (age, sex, ethnicity, knee OA duration, educational level, residential address and work status) were collected alongside anthropometric information (height and weight) to calculate body mass index (kg/m^2^) [28,29]. The Brief Pain Inventory – Short Form was used to collect baseline pain intensity [30].

Participants underwent 10–15 min of EMA training to aid in familiarising themselves with the smartphone, the EMA application (m-Path [27]), survey questions and to ensure that survey notifications were being received. Participants were either provided with a smartphone (Nokia 2.3, Nokia Corporation, using an Android 12 operating system (Snow Cone)) or could choose to use their own device and download the freely available m-Path application.

Participants completed 14 consecutive days of smartphone EMA monitoring (one wave; 10 weekdays and four weekend days) [12]. They were required to complete the smartphone survey three times daily [27,31]. EMA prompting occurred in a random-stratified manner, with participants being notified randomly within three pre-specified time blocks throughout their day. This ensured that symptoms after waking, during the day and in the evening were collected to get a representative dataset. The random-stratified blocks were scheduled as follows:

• Morning: A 2-h block was placed immediately following usual wake time.
• Day: A 5-h block was placed from 11 a.m. (or 2 h following usual wake time) [32].
• Evening: A 2-h block was placed immediately before usual bedtime.

Therefore, each participant was sent 42 surveys to complete during the study. Participants were required to complete their survey within 60 min, with responses >60 min being considered missing. Reminder prompting after 30 min as well as ‘snooze’ features for up to 60 min were also incorporated to improve compliance while reducing response bias [19]. Ordering differed between morning, afternoon and evening surveys with prior responses not being viewable. Relevant EMA survey items for the current analysis are included in Table 1.

**Table 1:** EMA survey items and schedule

EMA Survey Item	Morning	Day	Evening	Response
What is your level of pain right now?	✓	✓	✓	11-Point NRS (0 = No pain, 10 = Worst pain imaginable)
How much is your pain interfering with what you are doing right now?	✓	✓	✓	11-Point NRS (0 = No interference, 10 = Totally interfering)
How bothersome is your knee pain currently?	✓	✓	✓	5-Item ordinal scale (Not at all, slightly, Moderately, very Much, Extremely)
How severe is your knee stiffness currently?	✓	✓	✓	5-Item ordinal scale (None, mild, moderate, severe, extreme)

*Note.* NRS, Numeric Rating Scale.

The EMA survey administration and data collection were managed using m-Path [27]. Incoming EMA data were monitored, and participants were contacted if two consecutive assessments were missed. Reasons for loss to follow-up were recorded (n = 0).

### 2.4 Statistical analysis plan

For the current analysis, only the second week of the EMA data was used alongside the two-week follow-up KOOS. This was to ensure an alignment between the prospective EMA monitoring period and the KOOS recall timeframe (past week).

Descriptive statistics were performed to aggregate EMA variables. This included the calculation of means for current, maximum, minimum, variability and percentage of time in high or low symptoms. Means were also calculated for average levels of pain bothersomeness and knee stiffness. Relevant KOOS subscales and items were scored separately out of 100, where zero represented extreme problems and 100 represented no problems. Missing data trends for EMA data were analysed to determine potential patterns of missingness [33]. With data being deemed missing at random, mean imputation was performed as a robust and practical method to account for missing data [18,34].

To explore the relationship between EMA outcomes and KOOS outcomes, Pearson correlations (r) were calculated using SPSS (Version 29.0.2.0) [35]. Variables were assessed for normality by visually inspecting Q-Q plots. Non-parametric correlations (Spearman's rho) were performed when data were non-normally distributed and when single KOOS ordinal scale items were used (morning and evening stiffness). The overall strength of the relationship for each analysis was interpreted based on the following ranges: 0 to 0.09 – negligible, 0.10 to 0.39 – weak, 0.40 to 0.69 – moderate, 0.70 to 0.89 strong and >0.90 – very strong [36]. The level of error considered acceptable for statistical significance was set at p ≤ 0.05 with 95% confidence intervals also presented.

Partial correlations were calculated in SPSS (Version 29.0.2.0) to examine the influence of established demographic, anthropometric (BMI) and clinical prognostic factors for knee OA outcomes on these relationships [[37], [38], [39], [40], [41]]. Specific prognostic factors are chosen to explore the clinical relevance of the demonstrated relationships. To prevent model overfitting and maintain statistical power, a sensitivity analysis was conducted using G∗Power (Version 3.1) [42] to determine an appropriate number of covariates based on the sample size. Four covariates were deemed acceptable to maintain power at 0.8 for detecting a medium-sized effect [42]. Partial correlations controlling for age, sex, BMI, and baseline pain intensity were then computed, with all covariates entered into the model simultaneously alongside the independent and dependent variables [[37], [38], [39], [40], [41]].

## 3 Results

### 3.1 Participant characteristics

**Fig. 1:**
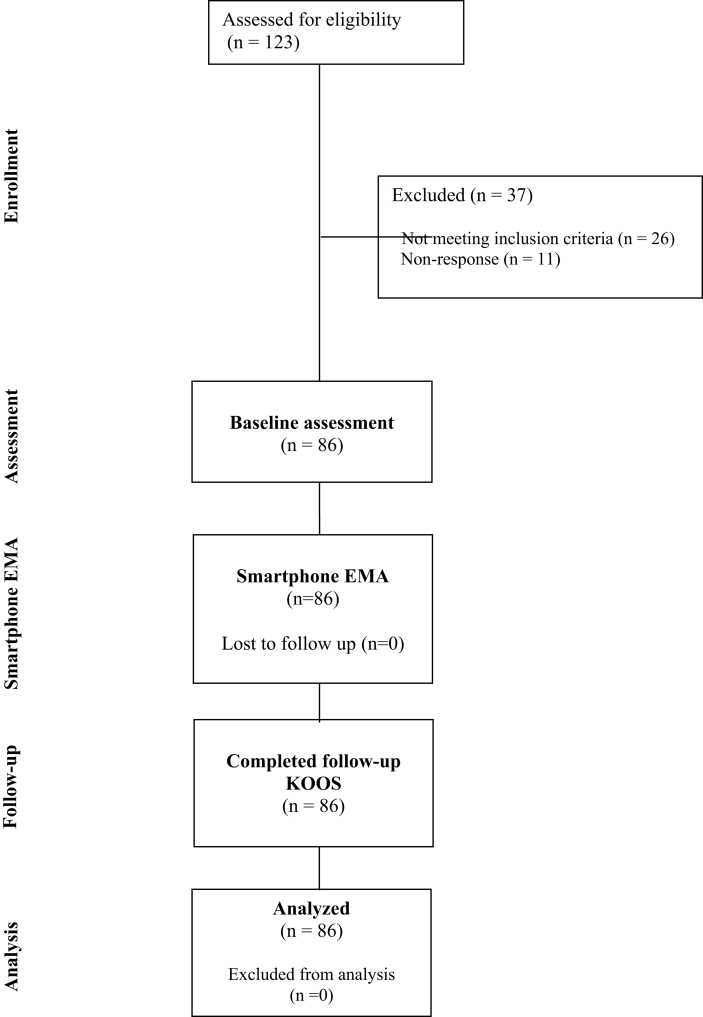
Participant flow diagram.

A final sample of 86 participants were included, with no loss to follow-up. The participant flow diagram can be seen in Fig. 1.

Characteristics of study participants are presented in Table 2.

**Table 2:** Characteristics of included participants (n = 86)

Characteristic	Value
Age [years; mean (SD)]	67.3 ± 9.1
Sex [n (%)]	Female: 55 (64)
	Male: 31 (36)
Ethnicity [n (%)]	NZ European: 78 (90.6)
	New Zealand Māori: 4 (4.7)
	Other: 4 (4.7)
BMI [kg/m^2^, mean (SD)]	32 ± 6.8
Knee OA duration [years; mean (SD)]	9.2 ± 9.1
Baseline KOOS [mean (SD)]	Pain: 55.6 ± 15.2
	Symptoms: 55.4 ± 18.1
	ADLs: 64.1 ± 18
	Sports and recreation: 36.2 ± 23.3
	QoL: 39.1 ± 19.7
Bilateral OA [n (%)]	Yes: 48 (55.8)
	No: 38 (44.2)
Worst knee [n (%)]	Right: 46 (53.5)
	Left: 40 (46.5)
Highest level of education [n (%)]	No formal qualification: 13 (15.1)
	Secondary school only: 12 (14)
	Trade/apprenticeship: 7 (8.1)
	Certificate/diploma: 19 (22.1)
	University degree: 19 (22.1)
	Postgraduate degree: 16 (18.6)
Work status [n (%)]	Full-time employed: 21 (24.4)
	Part-time employed: 9 (10.5)
	Self-employed: 7 (8.1)
	Homemaker: 1 (1.2)
	Retired: 47 (54.7)
	Unable to work: 1 (1.2)

*Note.* SD, standard deviation; n, number; BMI, Body Mass Index; kg, kilograms; m, metres; OA, osteoarthritis; KOOS, Knee Injury and Osteoarthritis Outcome Score; ADLs, activities of daily living; QoL, quality of life.

### 3.2 EMA participation and compliance

Average compliance during the second week of the EMA monitoring was 89.2%. Eighty participants (93%) used their own phones. All participants completed the study. Nonparametric correlations (Kendall Tau and Spearman) were completed to explore whether compliance was related to demographic variables. Only gender demonstrated a relationship with EMA compliance, with females responding to more of the smartphone EMA surveys (r = 0.3, p = 0.02). Non-response was deemed to be missing at random with no evidence of relationships between data missingness and clinical outcomes including pain intensity (p = 0.4), pain interference (p = 1), fatigue (p = 0.4), negative affect (p = 0.5), and the number of flare-up days (p = 0.9). There was no evidence of measurement reactivity with no statistically significant change in pain intensity ratings between weeks of EMA monitoring (p = 0.5).

### 3.3 Aggregated EMA measures

Aggregated data for Week 2 of the EMA are presented in Table 3 below.

**Table 3:** U-KOPE aggregated EMA data (week 2)

Pain intensity	Aggregated measure	Mean ± SD
	Current	2.8 ± 1.9
	Maximum	5.0 ± 2.4
	Minimum	1.3 ± 1.4
	Variability (SD)^1^	1.1 ± 0.5
	Time high (%)^2^	3.5 ± 12.5
	Time low (%)^2^	71.0 ± 34.1
Pain interference		
	Current	2.0 ± 1.7
	Maximum	4.5 ± 2.7
	Minimum	0.6 ± 1.0
	Variability (SD)^1^	1.1 ± 0.7
	Time high (%)^2^	2.0 ± 6.6
	Time low (%)^2^	72.9 ± 27.5
Bothersomeness		
	Current	1.16 ± 0.52
Stiffness		
	Current	1.16 ± 0.62
	Morning stiffness	1.15 ± 0.63
	Evening stiffness	1.16 ± 0.61

*Note.* U-KOPE, Understanding Knee Osteoarthritis Pain Experiences Study; EMA, Ecological Momentary Assessment; SD, Standard deviation; 1. Variability is the average of each participant's standard deviation. 2. Time in high (%) is the percentage of ratings ≥7.5/10. Time in low (%) is the percentage of ratings below ≤3.5/10.

### 3.4 Relationships between EMA outcomes and patient-reported outcomes (KOOS)

Statistically significant inverse relationships were demonstrated between all EMA outcomes and the KOOS subscales explored. The strength of relationships ranged from weak (EMA pain interference vs KOOS sport and recreation function - r = −0.38, p = <0.01) to strong (EMA pain bothersomeness vs KOOS pain – r = −0.78, p = <0.01) (Table 4).

**Table 4:** Adjusted and unadjusted relationships between EMA and KOOS outcomes

EMA item	KOOS subscale/item	Unadjusted correlation coefficient (95% CI)	p-value	Adjusted correlation coefficient^a^ (95% CI)	p-value
Pain intensity					
	KOOS pain	−0.69 (−0.79 to −0.56)	<0.01	−0.50 (−0.67 to −0.26)	<0.01
Pain interference					
	KOOS function (ADLs)	−0.62 (−0.73 to −0.47)	<0.01	−0.42 (−0.61 to −0.21)	<0.01
	KOOS function (sport and recreation)	−0.38 (−0.55 to −0.19)	<0.01	−0.25 (−0.44 to −0.08)	0.02
	KOOS function (combined)	−0.60 (−0.72 to −0.44)	<0.01	−0.41 (−0.59 to −0.19)	<0.01
Pain bothersomeness					
	KOOS pain	−0.78 (−0.85 to −0.68)	<0.01	−0.65 (−0.76 to −0.51)	<0.01
	KOOS function (ADLs)	−0.75 (−0.83 to −0.64)	<0.01	−0.61 (−0.78 to −0.40)	<0.01
	KOOS function (sport and recreation)	−0.45 (−0.61 to −0.27)	<0.01	−0.33 (−0.57 to −0.15)	<0.01
	KOOS function (combined)	−0.71 (−0.80 to −0.59)	<0.01	−0.57 (−0.75 to −0.35)	<0.01
Stiffness					
	KOOS stiffness (combined)	−0.61 (−0.73 to −0.46)	<0.01	−0.45 (−0.66 to −0.26)	<0.01
	KOOS stiffness (morning)^b^	−0.53 (−0.67 to −0.35)	<0.01	−0.35 (−0.53 to −0.14)	<0.01
	KOOS stiffness (evening)^b^	−0.58 (−0.70 to −0.41)	<0.01	−0.50 (−0.65 to −0.35)	<0.01

*Note.* EMA, Ecological Momentary Assessment; KOOS, Knee Injury and Osteoarthritis Outcome Score; CI, confidence interval; ADLs, Activities of Daily Living.

a Partial correlations adjusted for age, sex, BMI and baseline pain intensity.

b Spearman's rho correlation and partial correlation.

### 3.5 Adjusted relationships between EMA outcomes and patient-reported outcomes (KOOS)

Relationships between outcomes collected via smartphone EMA versus equivalent KOOS outcomes after controlling for age, sex, BMI and baseline pain intensity are presented in Table 4. Statistically significant inverse relationships were maintained between all EMA outcomes and the equivalent KOOS subscales explored. However, the strength of relationships changed with adjusted relationship strengths ranging from weak (EMA pain interference vs KOOS sport and recreation function – r = −0.25, p = 0.02) to moderate (EMA pain bothersomeness vs KOOS pain – r = −0.65, p = < 0.01).

## 4 Discussion

This study explored relationships between knee OA outcomes collected using a momentary smartphone survey versus those collected using a common, recommended PROM – the KOOS. Statistically significant relationships were demonstrated between both outcome measurement methods when collecting pain, function and knee stiffness outcomes. However, the strength of these relationships varied, ranging from weak to strong depending on the outcome domain assessed. After controlling for demographic and clinical factors involved in knee OA outcomes, statistically significant relationships were maintained; however, the strength of these relationships reduced.

Pain is a common symptom reported by those with knee OA [43]. When comparing momentary versus recalled methods for assessing pain in the current study, a moderate relationship was demonstrated. Potential reasons for only a moderate relationship being demonstrated include the differences in specific measures and the scale used to capture the domains of interest. For example, the activity-specific nature of items in the KOOS pain subscale, where pain ratings are collected on a Likert scale experienced during specific activities. In comparison with the smartphone EMA, participants were asked to report their current pain intensity on a numeric rating scale without reference to activity. Additionally, demographic and clinical factors influenced the strength of this relationship, with relationships becoming weaker after controlling for variables such as age, sex, BMI and baseline pain intensity. This highlights the influence of demographic and clinical factors in pain outcome assessment in people with knee OA.

A moderate relationship was also demonstrated between EMA and KOOS items measuring function. This included daily living as well as combined (daily living plus sport and recreation) function domains of the KOOS. The KOOS daily living function subscale asked participants to rate their degree of difficulty performing common daily tasks. Examples of activities include mobilising stairs, self-care, domestic tasks, as well as mobilising around the home and community. Meanwhile, the EMA survey asked participants to rate their degree of pain interference on a numeric rating scale during usual daily activities. Although the pain interfering with function (EMA-based) and difficulty in performing function are two different but overlapping constructs [44,45], they showed acceptable validity.

The strength of the relationship changed depending on the KOOS function subscale assessed. A weak relationship was demonstrated when comparing the KOOS sport and recreation function subscale with EMA, which further weakened after controlling for clinical and demographic factors. Participants in the current study were older adults (average age of 67 years) who had experienced knee OA pain for a prolonged duration (average of nine years). Therefore, the KOOS sport and recreation subscale might have been less relevant to capture the functioning of participants enrolled in this study [46]. Additionally, for the momentary measure, participants self-rated how pain interfered with their perceived ‘normal’ level of functioning rather than being forced to rate higher-level activities as required in the KOOS.

Pain bothersomeness is a novel pain outcome with links to pain intensity, disability, as well as unpleasant affective dimensions involved in the pain experience [18,47,48]. Interestingly, momentary reports of pain bothersomeness demonstrated the strongest relationships with KOOS outcome domains, even after controlling for established demographic and clinical factors influencing knee OA outcomes. The strength of the relationships between pain bothersomeness and KOOS pain, KOOS daily living functioning, as well as KOOS combined function were strong, while relationships between pain bothersomeness and KOOS sport and recreation function were moderate. Pain bothersomeness may be a single-item measure that better captures the biopsychosocial nature of knee OA pain [18]. These findings highlight the potential utility of monitoring pain bothersomeness in capturing pain as well as its psychological and functional impacts in people with knee OA.

Knee stiffness at specific times of the day demonstrated relationships when measurement methods were compared. The KOOS asked participants to rate knee-related stiffness in the morning and evening, while the smartphone EMA asked for knee stiffness ratings three times daily. When equivalent times were compared, the strength of the relationship was moderate. One potential reason this relationship wasn't stronger was that the EMA survey was scheduled to arrive randomly within 2 h of waking. A common characteristic of knee OA is that knee stiffness often reduces within 30 min of waking [49]. Therefore, the EMA survey may have missed ‘peak stiffness’ in comparison to recalled responses. When combining stiffness items to calculate KOOS average stiffness, the strength of the relationship increased. Average stiffness could therefore be a more balanced measure of knee stiffness in people with knee OA, which is likely to fluctuate throughout the day and be influenced by other symptoms such as pain [18]. Interestingly, controlling for knee OA prognostic factors had variable effects on the stiffness relationships explored. For morning and overall stiffness, the strength of each relationship reduced. In contrast, for evening stiffness the strength of relationship was maintained. This may reflect the unique variability in how people with knee OA rate their symptoms at different times of the day. Ratings in the morning may be more heavily influenced by clinical and demographic factors while evening ratings are more accurate and situational. The accuracy of symptom ratings over the day warrants further investigation.

Findings from the current study complement those reported in a recent systematic review and meta-analysis which explored relationships between momentary and recalled pain outcomes [11]. While this study is in general agreement, the strength of relationships between recalled PROMs and momentary outcomes was weaker overall. This may be explained by many of the studies in the systematic review using the same rating scale across different periods. Additionally, the activity-specific nature of the KOOS questionnaire may explain differences in relationship strength. For example, in the KOOS, individuals are asked to report pain or difficulties experienced during specific functional tasks. In contrast, EMA often includes single-item measures with participants rating current symptoms on a zero to 10 scale. This may result in a loss of nuanced, context-specific features of the pain experience in people with knee OA.

Cognitive biases have been reported as influencing perceptions of pain. These factors (which include recall bias) could contribute to differences in reporting between momentary and recalled PROMs [7,8,50]. By definition, pain is an unpleasant sensory and emotional experience [51]. Therefore, participants are more likely to recall and report easy-to-retrieve and emotive experiences such as episodes of high pain and greater functional limitation. Additionally, people with pain are more likely to recall more recent painful experiences (i.e. if they have experienced a flare-up over the last few days). These cognitive biases may influence perceptions of pain and functioning related to knee OA and skew ratings on recalled PROMs such as the KOOS. Alternatively, EMA methods are less susceptible to these biases as current symptoms are collected repeatedly in a momentary manner to avoid recall [15,50,52,53].

The current study does raise some considerations for outcome measurement and clinical practice. While smartphone EMA may more accurately capture variable and fleeting symptoms during daily life, this method is more burdensome and requires participants to have access to and the ability to use a smartphone. A recent qualitative study exploring the acceptability of smartphone EMA highlighted this. This study found that capturing pain experiences using this method was deemed acceptable by those with knee OA [54]. However, participants did note that overly burdensome EMA schedules (i.e. high number of daily assessments, prolonged timeframes) could impact their compliance and the overall acceptability.

Although PROMs are more susceptible to recall bias, they are more practical to administer. A person's recall is their reality, with recall shaping perceptions, coping behaviours and health decision making [55]. Recalled outcomes may therefore better reflect experiences of living with a painful condition (compared with what is actually ‘true’), making PROMs a more meaningful method of outcome measurement [56,57]. Therefore, rather than choosing a ‘best’ method for assessing knee OA, these methods could be viewed as complementary, with each providing slightly different information. For example, smartphone EMA increases ecological validity and captures difficulties experienced during a person's usual daily life. In contrast, PROMs are easily administered, less burdensome, and capture perceived limitations in specific functional tasks (even those avoided in daily life). Clinicians must therefore consider their goals with outcome assessment, the strengths and weaknesses of each method, and individualise decision-making to account for their patient and the clinical setting they work in.

Strengths of the current study included the study design adhering to the Checklist for Reporting Ecological Momentary Assessment Studies checklist to enhance methodological quality [19] and the high compliance of study participants without loss to follow-up. Additionally, knee OA outcomes were captured in momentary (or close to momentary) single-item questionnaires. This allowed these constructs to be collected in a manner that reduced burden and recall requirements. The survey items were piloted on a small group of study participants to inform the final EMA survey design [18]. Design features to reduce participant burden and improve data quality were also incorporated (e.g., training, individualised notification schedule, reminder/snooze features, use of own devices) to enhance the EMA experience. The strength of relationships was also assessed after controlling for demographic and clinical factors, which have been shown to influence knee OA outcomes.

The current study was not without its limitations. Only community volunteers who reported a mild-moderate knee OA presentation on average registered to participate. Despite attempts, no participants were recruited from the local hospital orthopaedic outpatient department, which would have increased the number of participants currently seeking treatment or with greater limitations due to their knee OA. Study participants also presented with a large degree of variability in knee OA duration. The presence of widespread pain in some participants as well as wording on the EMA pain items (i.e. aligning with BPI rather than knee-specific) may have impacted ratings and relationships. Additionally, the momentary, single-item measures often do not measure constructs in the same manner. Although the constructs/domains of interest (pain) are similar across the methods of data collection used in this investigation, the specific measures and metrics differ. For example, ratings of pain interference and KOOS function subscales measure different constructs, limiting the ability to make direct head-to-head comparisons. Additionally, the KOOS asked participants to recall symptoms or difficulties experienced during specific tasks, but the single-item measures are open to interpretation, with factors such as usual or ‘normal’ daily functioning influencing participant responses on numeric rating scales. Finally, although EMA captures symptoms in a momentary fashion and eliminates the need for recall, a true criterion standard is lacking.

Clinical implications based on findings from the current study include supporting the individualised tailoring of outcome measurement in people with knee OA. Rather than clinicians choosing one method over another, recall-based PROMs and EMA should be viewed as providing complementary information with clinicians deciding on best method based on their individual patients, their preferences, goals and health setting. Implications for future research include externally validating findings in larger samples across different locations, including populations with moderate-to-severe pain and functional restrictions associated with their knee OA. Additionally, establishing criterion validity of EMA measures for pain and function outcomes in other musculoskeletal pain conditions is required. Further investigation into the accuracy of measurements at different times of day is also warranted, as is exploration of the influence of contextual factors on the measurement accuracy of pain outcomes.

In conclusion, relationships were demonstrated between pain, stiffness and function outcomes collected via smartphone EMA and the KOOS. Although relationships were statistically significant, their strength ranged from weak to strong across the specific outcome domains assessed. Additionally, demographic and clinical factors influenced the strength of relationships. Stronger relationships were demonstrated between pain bothersomeness (measured using smartphone EMA) and KOOS domains assessing pain and daily functioning. In contrast, relationships with KOOS sport and recreation function were weaker. Therefore, this study supports the use of smartphone EMA and PROMs as potentially valid and complementary measures of pain, stiffness and function outcomes in people with knee OA.

## Author contributions

**MO**: Conceptualization, Methodology, Software, Formal Analysis, Investigation, Data Curation, Writing - Original Draft, Visualization, Project Administration, and Funding Acquisition.

**JD**: Conceptualization, Methodology, Writing - Review & Editing, Supervision.

**DS**: Conceptualization, Methodology, Writing - Review & Editing, Supervision.

**RM**: Conceptualization, Methodology, Formal Analysis, Investigation, Visualization, Writing - Review & Editing, Supervision, and Funding Acquisition.

## Role of the funding source

Funding for this project was provided by the Otago Medical Research Foundation Jack Thomson grant. The funder had no input into the research design, data analysis, interpretation and overall presentation of findings.

## Conflicts of interest

Nil identified conflicts of interest.
